# Next Generation DNA Sequencing and the Future of Genomic Medicine

**DOI:** 10.3390/genes1010038

**Published:** 2010-05-25

**Authors:** Matthew W. Anderson, Iris Schrijver

**Affiliations:** 1 Department of Pathology, Stanford University Medical Center, 300 Pasteur Drive, Room L235, Stanford, CA 94305-5627, USA; E-Mail: mwanders@stanford.edu; 2 Center for Genomics and Personalized Medicine, Stanford University Medical Center, 300 Pasteur Drive, Room L235, Stanford, CA 94305-5627, USA; 3 Department of Pediatrics, Stanford University Medical Center, 300 Pasteur Drive, Room L235, Stanford, CA 94305-5627, USA

**Keywords:** DNA, sequencing, next generation sequencing, bioinformatics, molecular diagnostics

## Abstract

In the years since the first complete human genome sequence was reported, there has been a rapid development of technologies to facilitate high-throughput sequence analysis of DNA (termed “next-generation” sequencing). These novel approaches to DNA sequencing offer the promise of complete genomic analysis at a cost feasible for routine clinical diagnostics. However, the ability to more thoroughly interrogate genomic sequence raises a number of important issues with regard to result interpretation, laboratory workflow, data storage, and ethical considerations. This review describes the current high-throughput sequencing platforms commercially available, and compares the inherent advantages and disadvantages of each. The potential applications for clinical diagnostics are considered, as well as the need for software and analysis tools to interpret the vast amount of data generated. Finally, we discuss the clinical and ethical implications of the wealth of genetic information generated by these methods. Despite the challenges, we anticipate that the evolution and refinement of high-throughput DNA sequencing technologies will catalyze a new era of personalized medicine based on individualized genomic analysis.

## 1. The first era of DNA sequencing: Sanger chemistry

In the late 1970’s, several groups described methods to chemically decode the composition of DNA utilizing either chemical cleavage of DNA [1] or incorporation of dideoxy-nucleotides during DNA synthesis [2]. In each instance, the radiolabeled products of the reaction were separated by size on a polyacrylamide gel and the DNA sequence was inferred by visually inspecting the banding pattern. A decade later, the advent of fluorescently labeled dideoxy-nucleotides [3] and automated capillary electrophoresis [4] enabled clinical and research laboratories to perform DNA sequence analysis on a routine basis. Indeed, DNA sequencing by these techniques (also termed “Sanger sequencing”) was later harnessed to sequence the entire human genome [5,6], and remains the mainstay of DNA sequence analysis for most laboratories. The mechanics of the technique are elegantly simple. First, the target DNA is amplified either by cloning into bacteria or by PCR. After purification of the template DNA, a primer is annealed adjacent to the sequence of interest and extended by DNA polymerase. During the extension reaction, the nascent chain is terminated by the random incorporation of fluorescently labeled dideoxy-nucleotides, which are complementary to the identity of the base on the opposite strand. Next, the reaction mixture containing fluorescently labeled DNA strands of varying length is resolved by capillary electrophoresis, and the resultant pattern of fluorescent peaks determines the DNA sequence. The technique is rapid, robust, has >99.9% raw base accuracy (the frequency in which the instrument correctly identifies a nucleotide from a known template sequence), and can typically achieve read lengths of up to 1 kb with relatively low cost. Therefore, Sanger sequencing is adequate for the majority of clinical applications involving the analysis of single genes with limited polymorphism. However, for many clinical applications such as the detection of somatic gene mutations in solid tumors and acute leukemia or the characterization of complex microbiological specimens, the level of sensitivity afforded by the Sanger technique (generally estimated at 10-20%) may be insufficient for detection of clinically relevant low-level mutant alleles or organisms. In addition, the analysis of highly polymorphic genomic regions such as the major histocompatibility complex (MHC) can generate complex electropherogram tracings secondary to multiple heterozygous positions in the sequence. During data analysis, the *cis* or *trans* orientation of heterozygous positions may be difficult to resolve, resulting in ambiguity of the allele assignment. Finally, the experience of sequencing the human genome [5,6] clearly demonstrated that the Sanger platform was not readily scalable to achieve a throughput capable of efficiently analyzing complex diploid genomes at low cost. Although some progress has been made to address these issues through high-density capillary array electrophoresis [7] and algorithms to deconvolute complex electropherogram tracings [8] these disadvantages are largely inherent to the technique.

## 2. Next generation DNA sequencing

The commercially available next generation sequencing platforms differ from traditional Sanger sequencing technology in a number of ways. First, the DNA sequencing libraries are clonally amplified *in vitro*, obviating the need for time consuming and laborious cloning of the DNA library into bacteria. Second, the DNA is sequenced by synthesis, such that the DNA sequence is determined by the addition of nucleotides to the complementary strand rather through chain termination chemistry. Finally, the spatially segregated, amplified DNA templates are sequenced simultaneously in a massively parallel fashion without the requirement for a physical separation step. While these advances are shared across all commercially available high-throughput sequencing platforms, each utilizes a slightly different strategy. In the following sections, we will detail the various high-throughput sequencing instruments commercially available. As the pace of this field is advancing quite rapidly, readers are referred to the manufacturers’ websites for the most current information regarding technical specifications and pricing. 

### 2.1. Roche/454 Life Sciences

**Figure 1:**
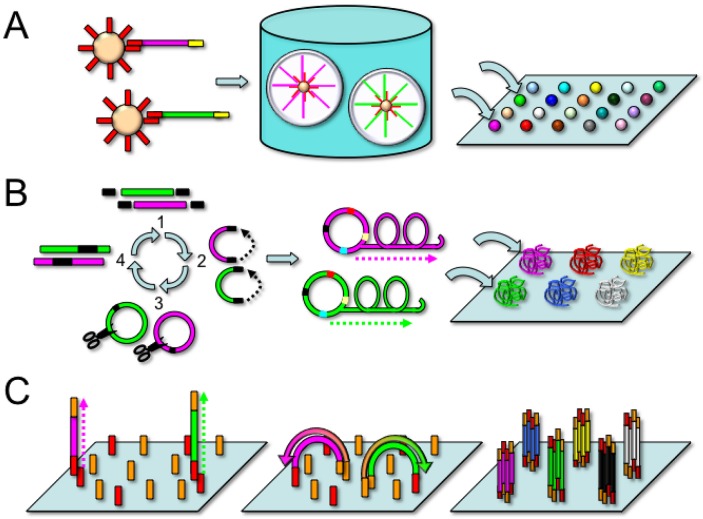
(A) Emulsion PCR. Adaptor sequences (red and yellow) are incorporated into the template DNA fragments (green and pink) through ligation or an initial PCR step. The adaptor sequences then hybridize to complementary capture oligonucleotides covalently linked to beads. The template molecules and beads are mixed together carefully to achieve an average of one template molecule per bead. Next, the beads are emulsified in an oil and water mixture, creating individual PCR microreactors for each bead/template combination. During emulsion PCR, the surface of the bead becomes coated with clonal copies of the DNA template. Next, the beads are deposited onto an array or microplate (arrows), such that each individual clonally amplified template is spatially segregated and sequenced separately. (**B)** DNA nanoballs. Genomic DNA fragments (green and pink) are ligated to adaptor oligonucleotides (black, step 1). The fragments are then circularized by ligating the adaptors together (step 2). Next, the circles are cleaved by restriction endonucleases (step 3), embedding the adaptor sequences within the template DNA (step 4). This process is repeated with the addition of new adaptor oligonucleotides (red, yellow, and blue) to produce a circular template with four embedded adaptor sequences to direct the sequencing reaction. Next, DNA polymerase is used to generate multiple linked copies of the template DNA (DNA nanoball), and the nanoballs are deposited onto the surface of an array in a spatially segregated fashion for sequencing. **(C)** Isothermal bridge amplification. Template DNA fragments (green and pink) are ligated to oligonucleotide adaptor sequences (orange and red), denatured to form single stranded DNA, and allowed to hybridize to complementary capture oligonucleotides covalently linked to the surface of the flow cell. Using the capture oligonucleotides as a primer, the templates are copied, and then denatured once again. The newly synthesized DNA molecules can then bend to hybridize with an adjacent capture oligonucleotide primer, which serves as the next primer for DNA synthesis. This process is repeated until clusters of multiple clonal copies of the template are generated on the surface of the flow cell.

In 2005, Jonathan Rothberg and colleagues reported the development of the first commercially available next-generation sequencing platform (454 Genome Sequencer) [9]. The first step of the 454 technique is the generation of a DNA library (single stranded DNA or PCR amplicons) containing flanking adaptor sequences which are used to immobilize the DNA library fragments to capture beads. Next, the adaptor-modified DNA library, PCR reagents, and capture beads are emulsified in a water-in-oil mixture to provide physical separation of the components into individual aqueous micro-reactors (Figure 1A). 

By adding the correct stoichiometric amount of the DNA library to the reaction mixture, one can ensure an average of one clonally amplified DNA molecule per bead. After amplification, the emulsions are broken with the addition of solvent, and the beads are enriched by incubation with streptavidin-coated magnetic beads to selectively purify beads containing biotin-labeled amplified product. A sequencing primer is annealed to the DNA bound to the beads, and the beads are loaded onto a fiber-optic “picotiter” plate containing millions of individual wells. To ensure one sequence read per well of the plate, each well has approximately the diameter of a single bead.

**Figure 2:**
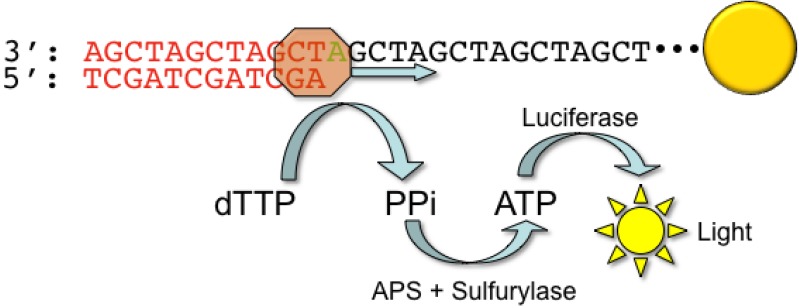
Pyrosequencing chemistry. DNA templates linked to a capture bead (yellow) are exposed to only one nucleotide during each round of sequencing. As a nucleotide (dTTP in this example) is incorporated through the action of DNA polymerase (brown), inorganic pyrophosphate (PPi) is released which reacts with adenosine 5’-phosphosulfate (APS) and sulfurylase to generate ATP. ATP is then used as a substrate for luciferase to generate light, which can be detected and quantified.

The 454 GS FLX instrument uses pyrosequencing technology to perform the sequencing reaction (Figure 2).

Originally described in 1996 [10,11], pyrosequencing takes chemical advantage of the pyrophosphate molecule liberated by the addition of a dNTP during the extension step. The pyrophosphate molecule is converted to ATP though the action of sulfurylase, and the ATP molecule is subsequently used by luciferase to convert luciferin to oxyluciferin. This reaction generates light, which can be measured and quantified by a highly sensitive camera within the instrument. For short single nucleotide repeat stretches, the intensity of the light emitted is proportional to the number of nucleotides incorporated. However, for longer homopolymer stretches (>8 nucleotides) the signal begins to show loss of linearity, with a concomitant rise in base call error rates. 

The key advantage of the 454 system when compared to other platforms is its longer read length and shorter run times. In eight hours, the second generation GS FLX instrument is capable of an output of 100 Mb with an average read length of 250 bases per template. Improvements to the picotiter plate and sequencing chemistry have increased the read length to an average of 400 bases with a corresponding increase in throughput (400-600 Mb). While the relatively low throughput results in the highest cost per base of the commercially available sequencing platforms, the long read length is critical for many applications including *de novo* genome assembly and detection of copy number variation. 

### 2.2. Applied Biosystems/SOLiD

**Figure 3:**
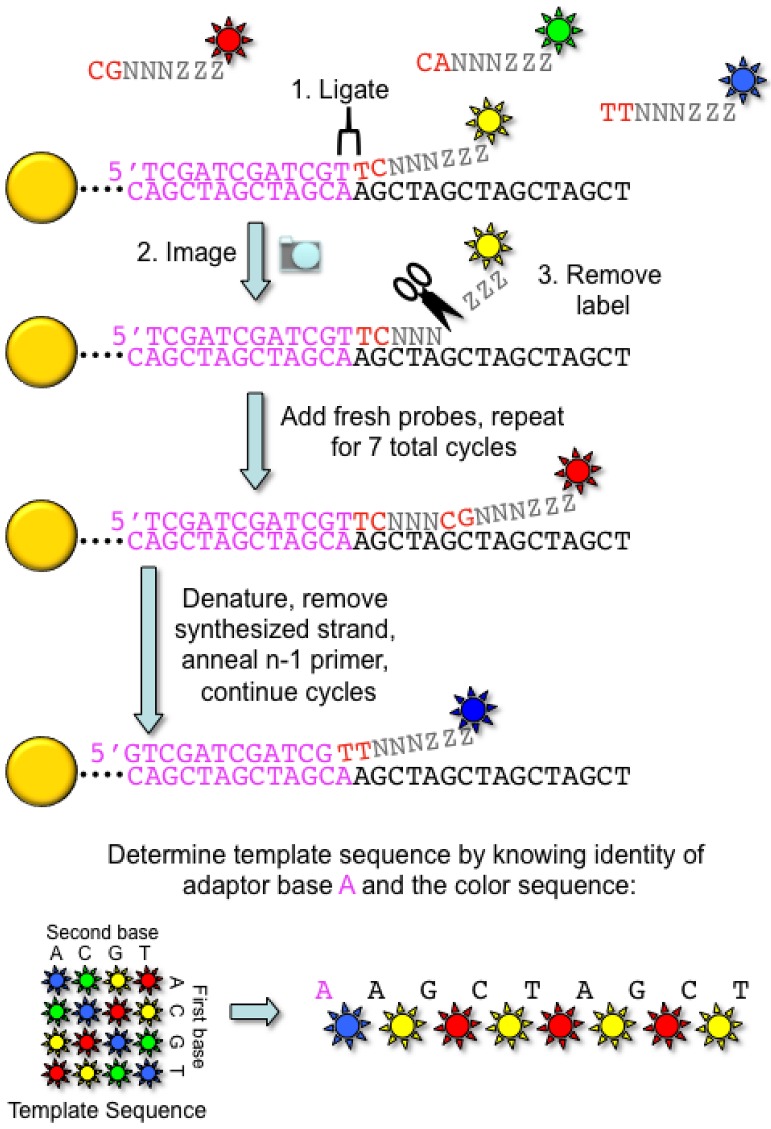
SOLiD ligation sequencing chemistry. DNA templates linked to a capture bead (yellow) are exposed to a mixture of sixteen different oligonucleotide probes encompassing all possible dinucleotide pairs (examples in red). The probes are fluorescently labeled with one of four colors, with each color representing four of the possible sixteen dinucleotide pairs. For example, the color blue represents the monodibase pairs AA, TT, CC, and GG. The remaining nucleotides in the probe are degenerate (NNNZZZ). After successful hybridization of a particular dinucleotide probe to the template sequence, the probe is ligated to the primer oligonucleotide, and the array is imaged. Next, the probe is cleaved, and the fluorescent label is washed away. This cycle of ligation, imaging, and cleavage occur for a total of seven cycles. Next, the newly synthesized strand is denatured and removed, and a new primer (offset by one base relative to the previous primer (n-1 primer)) is annealed to the template. The cycles of ligation, imaging, and cleavage continue for a total of seven cycles for each of 5 primers. The template DNA sequence is decoded by knowing the identity of the adaptor and the sequence of colors recorded from a particular template. As shown in this example, if the first nucleotide of the adaptor sequence is A (pink), and the first recorded color is blue, then the identity of the next base must be an A, as blue represents a monodibase pair. The remaining template sequence can then be deduced in a similar manner.

Originally developed in George Church’s laboratory in 2005 [12], the SOLiD technique differs from other commercially available high-throughput sequencing platforms in that the sequence is synthetically determined by a probe ligation method. Similar to the 454 approach, the first step is an emulsion PCR to generate a clonally amplified, adaptor-modified DNA molecule bound to a bead (Figure 1A). The 3’ end of the DNA template is modified to allow covalent attachment of the DNA beads to the surface of a coated glass slide within a flow cell. Next, a sequencing primer complementary to the adaptor sequence is annealed to the DNA template to provide a 5’ phosphate substrate for DNA ligase. To perform the sequencing reaction, fluorescently labeled 8-mer oligonucleotide probes are tested for the ability to anneal to the first two nucleotides of the DNA template immediately 3’ to the sequencing primer (Figure 3). 

The probes are constructed such that the first two positions represent each of the 16 possible dinucleotide combinations. The remaining six positions of the probe are degenerate and the 5’ end is labeled with one of four fluorescent labels. After annealing, DNA ligase covalently attaches the probe to the sequencing primer, and the fluorescence is recorded. The probe is then cleaved between positions 5 and 6, and the 5’ phosphate is regenerated to enable the subsequent ligation reaction. Seven cycles of these ligation reactions are performed. Next, the newly synthesized strand is denatured from the DNA template, and a new sequencing primer is annealed to the template. Importantly, the new primer is offset by one nucleotide relative to the initial sequencing primer (n-1). In total, the SOLiD instrument performs seven cycles of ligation from a total of five different sequencing primers, thus resulting in a read length of up to 35 bases. 

One of the advantages of the offset sequencing primer strategy is that each nucleotide in the sequence is interrogated twice. Therefore, a given nucleotide in the template sequence will generate two different fluorescent signals based on the identity of the neighboring base. The false positive rate for mutation detection is reduced, as a single nucleotide polymorphism (SNP) will generate two color changes when compared to the reference sequence. At the end of a six-day run, the SOLiD instrument is capable of generating 4 Gb of sequencing data. A related instrument developed by the Church laboratory (Polonator G.007) uses a similar oligonucleotide ligation approach to perform the sequencing reaction. The primary difference between the Polonator and the SOLiD platform is the reduced cost of the instrument and the open source nature of its software and analysis packages [13]. 

### 2.3. Complete Genomics

Complete Genomics (Mountain View, CA) has also developed an instrument that uses probe ligation chemistry similar to the SOLiD and Polonator platforms. However, instead of an emulsion PCR step, the DNA libraries are amplified as multiple copies of single stranded DNA termed “DNA nanoballs” [14]. In brief, restriction endonucleases are used to cleave the DNA templates, and then the resulting fragments are ligated together through the use of adaptor oligonucleotides to create circles of double stranded DNA. A polymerase then synthesizes hundreds of copies of linked single-stranded DNA (DNA nanoballs) from the circular template (Figure 1B). The DNA nanoballs are then hybridized to a patterned array containing over one billion individual spots. The ten template nucleotides immediately adjacent to the adaptor sequences are then interrogated using probe ligation sequencing chemistry. Utilizing this platform to sequence three HapMap individuals, the company reported an error rate of 1 false variant call per 100 kb, with a lower overall reagent cost than other commercially available high-throughput sequencing instruments [14]. Complete Genomics has no current plans to make their sequencing instrument commercially available, but it does offer in-house sequencing services bundled with web-based data analysis. This is an option for users who wish to perform whole-genome analysis without making the significant investment to purchase and maintain an instrument within their own facility.

### 2.4. Illumina Genome Analyzer

**Figure 4:**
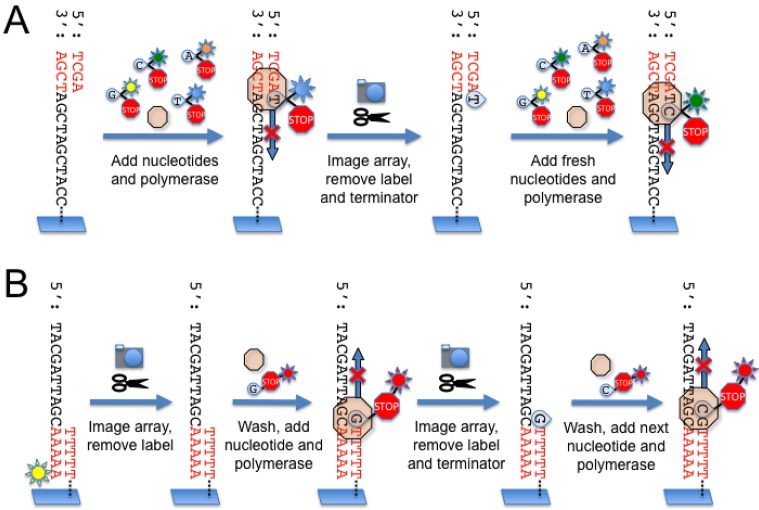
(A) Illumina sequencing chemistry. A sequencing primer (red) is annealed to the template molecules linked to the flow cell surface. Next, DNA polymerase and a mixture of fluorescently labeled nucleotides are added to the flow cell. The nucleotides are modified with a cleavable terminator moiety such that only one nucleotide can be incorporated during each sequencing cycle. After nucleotide incorporation, the array is imaged and the fluorescent signals are recorded for each cluster. The terminator moiety and fluorescent label are cleaved off and removed, and fresh nucleotides and polymerase are added to begin the next sequencing cycle. (**B)** Helicos sequencing chemistry. Template molecules modified by the addition of adenosines to the 3’ end are hybridized to poly-T oligonucleotides covalently linked to the surface of the flow cell. The template molecules are fluorescently labeled at the terminal 3’ adenosine so that the instrument can record the position of each template on the flow cell prior to the sequencing reaction. After the first image is acquired, the fluorescent label is removed and washed away. Next, DNA polymerase and one of four fluorescently labeled nucleotides (A, T, C or G) are introduced to the flow cell. Similar to the Illumina approach, the nucleotides are modified with terminator moieties to prevent multiple nucleotide additions during a single sequencing cycle. After nucleotide incorporation, the array is imaged and the fluorescent signals recorded. The fluorescent label and terminator moiety are removed, and the next cycle of sequencing commences with the next fluorescently labeled nucleotide.

The Illumina Genome Analyzer differs from both the 454 and SOLiD systems in that the clonal amplification step takes place *in situ* on the surface of the flow cell itself rather than in a separate emulsion PCR reaction. Similar to the other platforms, the DNA library is first ligated to oligonucleotide adaptors which incorporate a sequence complementary to “anchor” oligonucleotides which are covalently linked to the surface of the flow cell. After annealing to the anchor oligonucleotides, the template DNA molecules are clonally amplified in a modified isothermal PCR reaction termed “bridge PCR” [15,16], in which the DNA molecules are free to flex and form a “bridge” with an adjacent anchor oligonucleotide (Figure 1C). This process results in the generation of more than fifty million individual clusters containing over one thousand copies of clonally amplified DNA molecules on the surface of the flow cell. Next, the clusters are denatured to provide a single-stranded template, and a sequencing primer oligonucleotide is hybridized to the strand. During each sequencing cycle, the clonally amplified clusters are exposed to DNA polymerase and a mixture of four nucleotides, each labeled with a unique fluorescent label (Figure 4A). 

The nucleotides are modified at the 3’ end with a cleavable terminator moiety to ensure that only a single nucleotide incorporation event can occur with each sequencing cycle [17]. At the end of each cycle, the fluorescent signal is measured for each cluster, and both the fluorescent label and 3’ terminator moiety are cleaved and removed, regenerating the growing strand for another cycle of nucleotide addition.

Using this reversible terminator chemistry, the Illumina Genome Analyzer IIx instrument is capable of producing read lengths of 35 bp with >99% raw base accuracy and an overall throughput of approximately 5 Gb over a three day run. While the major source of error with this approach is incorrect incorporation of nucleotides, incomplete removal of either the fluorescent tag or terminator moiety also results in “dephasing” or asynchronous fluorescent signal generation between amplicons within a cluster. This imparts increasing “noise” to the fluorescent signal from a given cluster on the array, leading to a relatively poorer quality of base calls with longer read lengths. Ongoing improvements to the imaging system, sequencing chemistry, and analysis software may alleviate these issues and may allow for reliable increased read lengths [18]. 

### 2.5. Helicos

Originally developed by Stephen Quake and colleagues in 2003 [19], the Helicos system is unique among commercially available next-generation sequencing platforms in its ability to generate sequence information from non-amplified DNA templates. During sample preparation, genomic DNA is randomly cleaved to generate small fragments (100-200 bp). Next, multiple adenosines are appended to the 3’ end of the template molecules to allow the DNA templates to anneal to poly-T anchor oligonucleotides covalently linked to the surface of the flow cell (Figure 4B). The terminal adenosine is fluorescently labeled so the instrument can identify the position of each template molecule on the array prior to sequencing. The initial fluorescent label is cleaved and removed, and the sequencing cycles begin by exposing the templates to DNA polymerase and one of four fluorescently labeled nucleotides. Similar to the 454 approach, sequencing is asynchronous in that not all the templates will incorporate a nucleotide during a particular round of sequencing. After each round, the fluorescence signal is measured from each template by a highly sensitive fluorescence detection system. After hundreds of rounds of sequencing, the Helicos instrument can achieve an average read length of 30 bases and produce >20 Gb of sequencing data over a seven day run [20,21].

As there is no amplification step during sample preparation, the Helicos approach circumvents the problem of sequencing errors attributable to PCR artifacts. Like the 454 platform, errors may arise from multiple nucleotide incorporation events during sequencing of homopolymer regions. Recently, Helicos has introduced modified “virtual terminator” nucleotides [22], which prevent consecutive addition of nucleotides through a homopolymer region. Interestingly, the predominant sequencing error is a deletion, presumably due to incorporation of unlabeled nucleotides or due to detection errors. However, the overall accuracy of the technique is high (>99.99%), especially because the templates may be sequenced twice (two-pass sequencing).

## 3. ird generation” DNA sequencing

The ideal DNA sequencing platform would combine the advantages of high throughput, rapid sequence analysis with the capability to sequence long stretches of DNA. Long read lengths would significantly decrease the computational power required to perform genome assembly, detect genomic copy number variations, and provide important information as to the phase of allelic variants. Technologies currently under development include “reading” the nucleotide sequence directly by driving individual DNA molecules through a nanopore electrophoretically or by monitoring an individual polymerase molecule in real time as it synthesizes DNA. Although no “third generation” platform has been made commercially available as of yet, several companies have prototype technologies in active development [23,24].

### 3.1. Real time single molecule sequencing 

**Figure 5:**
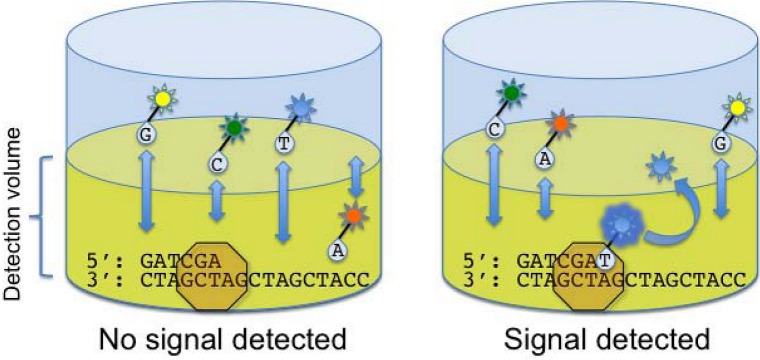
Single molecule real-time sequencing. In the SMRT technology developed by Pacific Biosciences, template molecules and DNA polymerase are immobilized at the bottom of an extremely small well termed a zero-mode waveguide (ZMW). The ZMW focuses the input energy from an excitation laser precisely to the zone containing the immobilized DNA polymerase, effectively reducing the detection volume. Nucleotides linked to different fluorophores through the terminal phosphate are then added. Unincorporated nucleotides pass rapidly in and out of the detection volume, too quickly for a measurable fluorescent signal to be recorded. However, when a nucleotide binds the active site of the DNA polymerase, its motion is sufficiently slowed for the fluorescent signal to be detected. As the nucleotide is incorporated, the fluorophore is cleaved off as the phosphodiester bond is formed. The free fluorophore then rapidly diffuses out of the detection volume, terminating the fluorescent signal for that particular nucleotide incorporation event.

Real time single molecule sequencing strategies attempt to “eavesdrop” on an individual DNA polymerase molecule in real time as it synthesizes DNA from a template strand. Given the highly processive nature of DNA polymerase, the read length would theoretically only be limited by the size of the DNA template molecule after sample preparation. However, novel biophysical and bioengineering solutions are required to accurately detect fluorescent signals generated during the relatively short timescale of nucleotide incorporation events catalyzed by DNA polymerase. Scheduled for commercial release in 2010 by Pacific Biosciences (Menlo Park, CA) the single molecule real time (SMRT) sequencer [24] segregates single polymerase molecules and DNA templates onto a plate containing thousands of nanometer-sized wells. The polymerase molecules are bound to the bottom of the wells and the optical system is finely tuned to measure fluorescence emitted from the bottom of the well, creating an extremely small (20 x 10^-21^ L) detection volume. The wells are then exposed to nucleotides that are fluorescently labeled via linkage to the phosphate. As a nucleotide is incorporated, it comes within the detection volume of the optical system, producing a fluorescent signal (Figure 5). 

Next, the polymerase continues to the next position and the fluorescent moiety is cleaved from the growing strand. It then quickly diffuses out of the detection volume. Because the synthesized DNA strand is composed entirely of “natural” DNA bases, the efficiency of DNA polymerase is not adversely affected by the steric effects of modified nucleotides. From the limited published data on this technology [24], SMRT appears to have the capacity to achieve read lengths of greater than 2000 nucleotides, with a median base accuracy of 99.3% when 15-fold coverage of a given sequence is attained. To enable repetitive sequencing, template DNA fragments can be ligated to hairpin oligonucleotides, creating a circular DNA template which can be repeatedly sequenced in a single well. The SMRT instrument can also directly detect methylated nucleotides by measuring alterations in polymerase kinetics [25], enabling simultaneous analysis of both the primary DNA sequence and methylation status during a single sequencing run. Life Technologies (Carlsbad, CA) has recently unveiled a single-molecule sequencing instrument that utilizes fluorescence resonance energy transfer (FRET) from a quantum-dot labeled polymerase to a labeled nucleotide. While a FRET-based approach may theoretically result in lower base call error rates, detailed performance metrics of this technology are not yet available. 

### 3.2. DNA sequencing by direct physical methods

Spurred by the Archon X genomics prize for sequencing 100 human genomes in 10 days for less than $10,000 per genome [26], a few groups have proposed alternative sequencing methods that determine the sequence of the DNA template by the distinct chemical and/or physical properties of each nucleotide without the use of polymerase or fluorescent labels. Theoretically, directly reading the DNA sequence by an electrochemical and/or physical approach would likely be faster and more cost-effective than all the technologies yet developed. Various approaches have been proposed, including electrophoretically driving DNA through nucleotide-sensing nanopores [23], and directly visualizing DNA molecules by electron microscopy. These technologies could have the added benefit of being able to directly sequence RNA as well as DNA. However, difficult engineering challenges must be overcome before these technologies could become commercially viable. As such, these technologies are currently limited to the research and development setting.

## 4. Genomic enrichment strategies

Although whole-genome sequencing analysis may be soon feasible for the clinical laboratory from a technical perspective, targeted analysis of specific genomic regions may be preferable in order to answer a specific clinical question. For example, in bone marrow and solid-organ transplantation, a complete analysis of the genes within the MHC for both donor and recipient may provide critical information as to the potential for organ rejection or graft failure post-transplant. For a patient with cancer, an oncologist may wish to perform rapid mutation screening of a variety of genes encoding proteins (such as tyrosine kinases) that are targets for therapeutic agents. Therefore, a robust method is needed to enrich specific genomic regions prior to high-throughput sequencing. In recent years, several approaches have been developed to enrich for the protein-coding regions of the genome (exome) [27] using modified multiplex PCR [28,29], capture by circularization [30], or capture by hybridization in solution or on an oligonucleotide array [31]. In a recent demonstration of the power of this approach, Ng *et al.* used array-based exome enrichment and high-throughput sequencing to identify the gene involved in Miller syndrome, a rare mendelian disorder [32]. Although exome-based strategies help narrow the search for causative genetic loci, these technologies do not detect sequence variants within non-coding regions. In addition, each technique is subject to different selection biases specific to the particular capture technology. With the advent of single-molecule sequencing instruments that can sequence long stretches of DNA in-phase, novel genomic enrichment strategies will have to be developed to also allow for the capture of larger intact DNA fragments. 

## 5. Data processing

Although many clinical molecular pathology laboratories have staff with the technical expertise to adapt to performing high-throughput sequencing, the overwhelming amount of sequence data generated from a single patient specimen creates new challenges for the laboratory, requiring significant investment in bio-informatics infrastructure and personnel with programming expertise, if the computational analysis is to be done in-house. Although each next-generation sequencing platform has a unique data processing pipeline, similar strategies are used to transform the raw sequence data into a form amenable to interpretation. First, as millions of sequencing reactions are occurring in parallel, one must first analyze global run performance metrics to ensure that the instrument (plate, reagents, *etc.*) is performing within specification. To accomplish this, many of the next-generation sequencing instruments include within-run standard control sequences. Next, each individual sequencing read must undergo a quality assessment designed to address the error modalities commonly observed with a particular sequencing chemistry. For example, software algorithms have been developed to mitigate the “dephasing noise” which occurs toward the end of Illumina reads [18], and to define criteria to identify deletion or insertion errors which occur in homopolymer regions during 454 pyrosequencing [33].

After the sequences have undergone quality assessment, the genomic sequence must be “re-created” either through alignment to a reference genome or *de novo* assembly. While alignment to a reference genome may be simpler to perform in terms of computational effort, the -at least currently- relatively small number of reference human genomes may hamper unbiased detection of SNPs and structural variations in a patient specimen. To perform efficient alignment of short-read sequence data to a reference genome, a variety of computational methods have been developed (reviewed in [34]). The two most common strategies are either to convert the sequence data (or the reference genome) into a series of unique integer values (Hash tables), or to perform a Burrows-Wheeler transform to construct a matrix of all possible rotations of a given sequence. To perform *de novo* genomic assembly, long stretches of DNA sequence must be created from the shorter read length data. With Sanger technology, the relatively long read length allows for sequence assembly based on the degree of overlap between sequencing reads. However, this approach is not computationally feasible for the short read lengths produced by next-generation sequencing systems. To solve this problem, new algorithms were developed which analyze the sequence data as small fixed-length sub-sequences [35]. These algorithms have been incorporated into software programs, one of which (ABySS) has been used to perform successful *de novo* whole-genome assembly of a Yoruban individual [36]. With the advent of longer-read high-throughput sequencing technologies, the computational power required to perform *de novo* genomic assembly will likely decrease with a concomitant improvement in variant detection.

## 6. Applications of next-generation sequencing for clinical diagnostics

**Figure 6:**
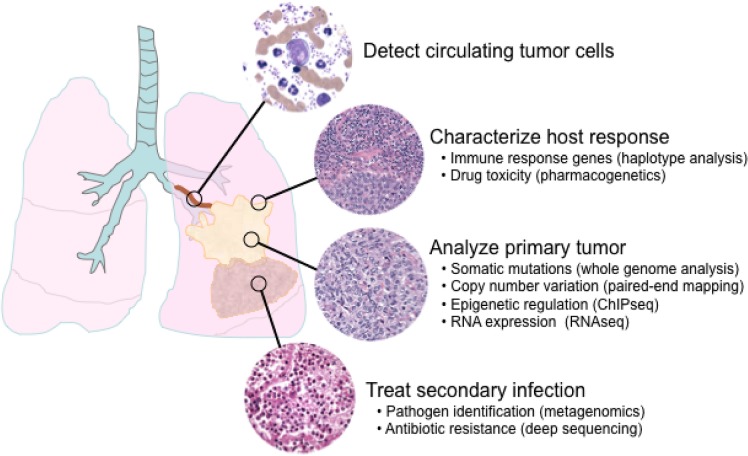
Possible applications of next-generation sequencing for clinical diagnostics. In this hypothetical clinical scenario, a patient presents with carcinoma of the lung and an associated post-obstructive bronchopneumonia. Diagnosis by traditional morphologic analysis of pathologic material will be complemented by high-throughout sequencing assays to analyze the tumor on a molecular level. Patient prognosis and response to therapy will be more precisely defined by high-throughput sequencing assays to characterize the host response to the tumor and to detect tumor cells in the peripheral blood. Complications such as a concomitant infection can be more accurately diagnosed and managed. In the future, the ability to integrate pathologic, clinical, and genomic data as shown in this example is expected to result in improved diagnosis and treatment for patients.

The development of high-throughput sequencing technologies has enabled research laboratories to investigate disease mechanisms from the DNA sequence to transcriptional regulation and RNA expression. As complex diseases are likely secondary to global perturbations in cellular and physiologic networks, integrated reporting of analyses including DNA sequence variants, RNA expression levels, and promoter methylation status may become increasingly relevant for diagnosis and for prediction of response to therapy. For the clinical laboratory, the challenges of expanding into these new areas of nucleic-acid testing are daunting, and will likely require the use of multiple complementary high-throughput sequencing technologies. In this section we will briefly describe some of the possible applications of next-generation sequencing technology for clinical diagnostics (Figure 6).

### 6.1. Single nucleotide polymorphisms and somatic mutations

Understanding the relationship between DNA variation and disease has long been a major focus of human genetics research. However, the identification of specific genetic loci underlying complex diseases remains challenging. One approach is to catalogue genetic variation (SNPs) across the genome and attempt to associate those variants with a particular phenotype (genome-wide association or GWA) [37]. To date, high-density oligonucleotide arrays have been the predominant methodology for SNP genotyping in large-scale collaborative efforts such as the International HapMap Consortium [38,39]. However, the ability to detect SNPs using array-based approaches is limited by the density of the array [40]. As high-throughput sequencing technologies provide single nucleotide resolution, rare variants can now be detected and characterized [41,42], including mosaic mutations [43]. A database of sequence variants that were discovered using high-throughput sequencing is currently being created as part of the 1000 genomes project [44]. Indeed, the power of high-throughput sequencing to identify unknown causative mutations in human disease has recently been demonstrated in a family with a recessive form of Charcot-Marie-Tooth disease [45], and in a family with both Miller syndrome and primary ciliary dyskinesia [46]. Comprehensive SNP identification will undoubtedly improve the predictive power of GWA studies, and likely impact our understanding of complex disease trait loci and pharmacogenomics. 

The improved detection of rare sequence variants by high-throughput sequencing can also be applied to the discovery of novel somatic mutations in cancer. Recently, several groups have performed comprehensive genomic analysis of a variety of cancers including acute myeloid leukemia [47,48], lung cancer [49], and melanoma [50]. These efforts have catalyzed a collaborative research effort (International Cancer Genome Consortium [51]), which will collect data from hundreds of individual samples of fifty different cancer types. These data are expected to lead to a better understanding of the molecular pathogenesis of cancer, and will undoubtedly result in novel diagnostic and therapeutic approaches. 

### 6.2. Haplotype analysis

Haplotype analysis refers to determining whether two sequence variants are present on the same copy of a chromosome (in *cis*), or on opposite chromosomes (in *trans*). For monogenic autosomal recessive disorders, the phenotype is critically dependent on the *cis* or *trans* orientation of a particular combination of pathogenic mutations. The linkage of consecutive SNPs along a particular chromosomal region facilitates GWA studies, and can elucidate the evolutionary history of human populations [52]. For highly polymorphic gene regions such as the MHC, multiple heterozygous positions complicate haplotype analysis, resulting in ambiguities in the final human leukocyte antigen (HLA) genotype. Such ambiguity may have serious clinical ramifications. For example, in bone marrow transplantation, ensuring an accurate HLA match between donor and recipient is critical to promote engraftment and to reduce the risk of graft *versus* host disease [53].

The *cis/trans* distinction is often difficult to assess using Sanger sequencing protocols in which both chromosomal complements are amplified and sequenced together. The traditional solution to phase determination has been through cloning PCR products into bacteria, but this approach is laborious and time-consuming. High-throughput sequencing protocols offer a clever way to avoid bacterial cloning through an *in vitro* clonal amplification step. Template DNA molecules are spatially separated during amplification by either oil-in-water microreactors (454, SOLiD) or by hybridization to surface-linked oligonucleotides (Illumina) (Figure 1A and 1C). However, even with a clonal sequencing template, the correct phase assignment can only be made if two sequence variants are present within the read length of a particular sequencing chemistry. Taking advantage of *in vitro* clonal amplification by emulsion PCR and the longer read length afforded by pyrosequencing chemistry, several groups have used amplicon sequencing with the 454 platform to address the phase problem for HLA genotyping [54,55]. These studies demonstrated good concordance between the HLA genotyping results from 454 pyrosequencing and traditional Sanger-based sequencing without the need to perform additional testing to resolve phase ambiguities within the analyzed regions. Indeed, with the development of automated methods to template preparation and emulsion PCR, the 454 approach to HLA genotyping may soon be amenable to routine use in the clinical histocompatibility laboratory. However, newer single-molecule sequencing instruments may eventually offer a more efficient solution to the issue of phase ambiguity by sequencing kilobases (or more) of DNA in phase from a single template. 

### 6.3. Copy number variation

Although much attention has been paid to the detection of SNPs, copy number variation (CNV) of DNA segments comprises a significant amount of the genetic variation amongst individuals [56,57]. CNV has also been implicated in diseases including psoriasis and autism [58]. Many of these studies were conducted through the use of array-based comparative genomic hybridization. While array-based approaches can detect large CNVs (with a resolution of approximately 1 kb), they cannot detect balanced structural variations such as inversions [59]. High-throughput sequencing can be used to detect balanced and unbalanced CNVs through a technique called “paired-end mapping”. In this approach, genomic DNA is sheared to a defined size and ligated at each end to adaptor oligonucleotides. The adaptors are then ligated to each other to form a circularized fragment of DNA. After an additional fragmentation step, the genomic DNA adjacent to the adaptors is sequenced, and the sequences are mapped to a reference genome. In a demonstration of this approach using 454 technology, Korbel *et al.* detected deletions, inversions, and insertions with an average resolution of 644 bp [60]. Paired-end mapping has also been used with the Illumina platform to detect somatic rearrangements in lung cancer [61] and breast cancer [62]. Although sequencing-based approaches to detect CNV are currently too expensive and laborious for routine clinical diagnostics, longer read lengths and lower reagent costs may, in the future, enable sequencing techniques to replace array genomic hybridization in the clinical laboratory.

### 6.4. Epigenetics

In recent years, there has been a greater appreciation of how epigenetic regulation of gene expression underlies the pathogenesis of many diseases, especially cancer [63]. Perhaps the best understood mechanism of epigenetic regulation is the reversible methylation of cytosine residues located within CpG repeat sequences. CpG repeats are frequently located in the promoter regions of genes, and methylation of these regions leads to a cascade of protein-binding events resulting in chromatin remodeling and transcriptional repression. In cancer cells, aberrant methylation can silence genes that are important for orderly cell division (for example genes encoding DNA repair enzymes or p53) and can promote tumor progression. Indeed, diseases including myelodysplastic syndrome [64] and colorectal cancer [65] have been linked to aberrant methylation. Methylation status may also be useful to predict response to chemotherapeutic agents [66]. With the advent of pharmacological agents that can demethylate and thus reactivate repressed genes, there is increasing clinical interest in the detection and quantification of methylation status. As methylation involves direct modification of a nucleotide, sequencing-based approaches can detect both the presence and the location of a methylation event. Sodium bisulfite conversion (which converts unmethylated cytosines to uracil) followed by high-throughput sequencing has been used to describe genome-wide methylation patterns in mouse embryonic stem cells [67], and in human breast cancer [68]. In addition to identifying which genes are methylated in a particular disease state, these techniques may be useful to select patients for demethylation therapies and to monitor the therapeutic response to these agents [69]. 

Another important mechanism of epigenetic regulation is through DNA-binding proteins such as transcription factors and histones. DNA sequences bound to a particular DNA-binding protein can be determined experimentally by a technique termed chromatin immunoprecipitation (ChIP) [70]. The procedure involves the chemical cross-linking of DNA-protein complexes, fragmenting the DNA, and isolating the DNA-protein complexes by immunoprecipitation with an antibody specific to the protein of interest. Currently, the most widely used technique to identify genes that are affected by protein binding is to hybridize the eluted DNA molecules to oligonucleotide arrays (ChIP-chip) [71]. In an effort to increase sensitivity, specificity, and genomic coverage of this technique, high-throughput sequencing has also been used to analyze the eluted DNA molecules (ChIP-seq) [72]. ChIP-seq has been used to characterize histone and transcription-factor binding sites in human CD4^+^ T cells [73], a cervical carcinoma cell line [74], and pluripotent murine stem cells undergoing differentiation [75]. While high-throughput sequencing has improved our ability to detect and characterize DNA-protein interactions, further work is required to determine how these dynamic changes result in a defined clinical disease phenotype.

### 6.5. Transcriptome analysis

Global analysis of RNA expression can enhance our understanding of both normal cellular physiology and disease states. Indeed, one of the hallmarks of cancer is aberrant mRNA expression, which often directly reflects abnormal cellular processes such as de-differentiation, resistance to apoptosis, increased proliferation, and propensity to metastasis [76]. Over a decade of research aimed towards understanding the role of RNA expression in cancer has led to a more complete molecular description of the biological networks common to carcinogenesis across different histological subtypes of cancer [77]. Many of these discoveries have resulted in the development of clinical assays to predict prognosis and to guide therapy, most notably in breast cancer [78,79]. The rapid pace of RNA profiling in cancer has been due, in large part, to the development of DNA microarray technology [80,81]. However, microarray technology is limited in that transcript abundance is measured indirectly through hybridization, and each probe is targeted to a small portion of the gene. This imparts noise to the data, makes the comparison of expression data across array platforms and experiments difficult, and complicates the use of the data for biomarker discovery [82].

Given the limitations of DNA microarray technology, high-throughput sequencing approaches have been adapted to perform whole transcriptome analysis (RNA-seq) [83]. In a typical RNA-seq experiment, total RNA or poly-A selected RNA is isolated, cDNA is generated, and the cDNA is fragmented and ligated to adaptor sequences to provide templates for high-throughput sequencing. As these experiments essentially count transcript abundance, they are an ideal application for high-throughput sequencing instruments with short read lengths. Numerous variations of RNA sequence analysis have been developed, including protocols to measure RNA expression from difficult specimens such as paraffin-embedded tissue [84]. 

The ability to analyze the transcriptome at single nucleotide resolution has transformed our understanding of RNA expression in human biology and disease. RNA-seq has been used to characterize the transcriptome of human B-cell and kidney lines [85], and a cervical cancer cell line [86]. RNA sequence analysis has also been used to detect gene fusions in prostate cancer [87], and to discover novel somatic mutations in tissue samples from patients with granulosa cell tumors of the ovary [88]. In addition to mRNA, small non-coding RNAs such as microRNAs have been analyzed in various tumor types [89-91]. Finally, transcriptome profiling has been performed on microbial pathogens relevant to human disease such as *Helicobacter pylori* [92]. With recent large-scale projects to characterize the human mRNA transcriptome in healthy HapMap subjects [93,94], our ability to relate changes in the transcriptome to disease phenotype will continue to improve.

### 6.6. Metagenomics and minimal residual disease detection

The ability to detect and quantify small numbers of infectious organisms or circulating tumor cells is clinically useful to direct therapy and predict patient prognosis. To date, the most commonly used method for sensitive nucleic acid detection in the clinical molecular diagnostic laboratory is quantitative PCR (qPCR). While qPCR assays are highly sensitive and specific, they require *a priori* knowledge of the target sequence. In contrast, next-generation sequencing is an unbiased approach to nucleic acid detection. Coupled with the immense numbers of individual sequence reads produced by high-throughput sequencing instruments (deep sequencing), next-generation sequencing instruments offer a novel approach to detect infectious organisms and minimal residual disease (MRD).

As many clinically relevant micro-organisms are difficult to culture, infectious disease testing in the clinical laboratory has increasingly relied upon molecular diagnostic techniques [95]. The combination of high-throughput clonal template amplification and deep sequencing enables identification of multiple, potentially novel species from a complex microbial mixture without the use of culture techniques (metagenomics) [96]. This approach has been used to identify novel viral pathogens [97], detect viral drug-resistance mutations [98,99], and diagnose bacterial infections [100]. However, given the relatively high cost of high-throughput sequencing, these techniques are unlikely to replace traditional microbiological techniques for routine pathogen identification in the immediate future. 

MRD detection is important for many diseases including leukemia and lymphoma [101] and the detection of small numbers of circulating tumor cells (CTC) may be an important predictor of prognosis in patients with solid-organ malignancies [102]. Designing clinical assays to detect MRD or CTC by molecular methods is relatively straightforward if the nucleic acid target is similar for a majority of patients with a given disease. For example, the *BCR-ABL1* gene rearrangement characteristic of chronic myelogeneous leukemia exhibits a few common breakpoints, each of which can be detected by qPCR to monitor molecular response to tyrosine kinase inhibitor therapy [103]. However, for diseases with heterogeneous molecular defects, MRD or CTC detection using qPCR techniques requires designing and validating a unique primer set tailored to each individual patient. High-throughput sequencing methods can improve MRD detection by characterizing genomic alterations specific to a given patient’s tumor, or through deep sequencing to detect small amounts of mutant or clonal DNA without *a priori* knowledge of the mutant DNA sequence. In an example of the first approach, Leary *et al.* [104] used mate-pair library sequencing on the SOLiD platform to characterize patient-specific translocations in solid-organ tumors, and then designed custom digital PCR assays to quantify the number of rearranged DNA molecules circulating in the patient’s plasma. In an elegant demonstration of the latter approach, Boyd *et al.* [105] used the 454 platform to characterize B cell repertoires in normal patients and detect small numbers of clonal B cells in patients with B cell lymphomas. Coupled with the use of barcoded amplicon primers to multiplex multiple patients in a single run [106], this approach may become one of the first applications of high-throughput sequencing to be adopted by clinical molecular laboratories.

## 7. Whole genome analysis and clinical diagnosis

Practiced most efficiently, clinical diagnosis is an iterative process that begins with the patient history and physical examination to generate a focused differential diagnosis [107]. Laboratory and imaging studies are then selected to help guide hypothesis testing and narrow the diagnostic possibilities. Subsequently, appropriate additional diagnostic tests are ordered, as necessary, in a logical and sequential manner. For example, in the pediatric genetics clinic, the patient’s history, physical examination, family history, imaging studies, and laboratory results are all carefully reviewed and integrated prior to selecting one or a few likely gene candidates to examine, at the DNA sequence level, as the possible cause of the patient’s symptoms. While this time-honored method of practicing clinical medicine is not always strictly adhered to, this approach limits diagnostic bias and is largely cost effective. However, as we enter an era in which whole-genome sequence analysis becomes more realistically possible to consider for clinical laboratory applications, the ability to interrogate the genomic sequence of an individual patient poses a major challenge to the traditional practice of medicine. In effect, the diagnostic process may shift from iterative hypothesis testing to inferring causality from sequence variations in genes linked to a disease-associated physiologic pathway. Without careful consideration of the limitations of whole-genome analysis, genomic “fishing expeditions” could have serious adverse consequences for patients, both physically and psychologically. Issues surrounding whole-genome analysis are complex, and will require collaboration among physicians, ethicists, genetic counselors, patients, and other stakeholders in the health care system. In the following sections, we will discuss some of these issues and identify possible benefits and pitfalls to implementing whole genome analysis in routine patient care.

### 7.1. Accuracy

The accuracy of a laboratory technique can be broadly defined as the ability to reproducibly generate a result reflecting an underlying biological “truth”. For clinical molecular diagnostic laboratories, the accuracy of DNA sequence analysis encompasses at least three components. First, the technical component of the assay (*i.e.* capillary electrophoresis) must have sufficient sensitivity and specificity to ensure correct and reproducible detection of sequence variations. Second, the software used to analyze the sequence data must also be evaluated for its ability to detect and report sequence variations. Finally, the laboratory must report the results using standardized nomenclature, and provide current and accurate interpretation of the significance of a given sequence variation.

Quality control for Sanger sequencing technology is relatively straightforward, typically requiring the analysis of control DNA of a known sequence. The quality of the sequence can be determined by both visual inspection of the capillary electrophoresis tracings and an assessment of the signal to noise ratio. Due to the large number of sequences generated by next-generation sequencing instruments, however, direct visual inspection of each individual sequencing result is not feasible. Therefore, the user must rely on quality metrics generated by the instrument itself to determine the overall quality of a given run. Because next-generation sequencing chemistries have a higher intrinsic error rate than Sanger sequencing, each template may have to be sequenced multiple times to mitigate errors. For example, a clinical laboratory using high-throughput sequencing for MRD detection may experimentally determine a minimum threshold for the depth of sequence coverage required to reproducibly detect rare sequence variants. However, a clinical laboratory cannot possibly design a validation to ensure that a particular high-throughput sequencing platform can reliably detect all possible sequence variants in diseases with unknown genetic cause. Confirmation of novel sequence variants detected by high-throughput sequencing will require additional costly and time consuming testing by other techniques. Therefore, we expect that the first uses of high-throughput sequencing technology will be targeted to limited genomic regions or genes for which “gold-standard” assays are already available.

Establishing the sensitivity, specificity, and reproducibility of high-throughput sequencing assays in the clinical laboratory will pose a challenge to the implementation of these technologies. The validation process for molecular genotyping assays (even those targeting a single SNP) is complex, and requires significant laboratory investment in both time and resources [108]. Validating a high-throughput sequencing instrument for clinical diagnostics becomes significantly more challenging when one considers both the reagent cost and technical as well as computational expertise required. For example, establishing performance metrics across multiple independent sequencing runs could become prohibitively expensive. For the near future, collaborative efforts among manufacturers and clinical laboratories may help mitigate the high start-up costs for early adopters, and improve the design and use of these technologies in clinical diagnostics. 

### 7.2. Genotype/phenotype correlation

Few would question that our technical ability to interrogate thousands of genes using high-throughput sequencing has far outpaced our skill to interpret the data in a clinically meaningful way. Genotype/phenotype correlation is immensely difficult even for single-gene disorders, and requires in-depth knowledge of how a particular sequence variant may affect a number of biological events including gene regulation and protein function. Although a nonsense or frameshift mutation is likely pathogenic, there are examples in which understanding the clinical phenotype depends on knowledge of nuances of the encoded protein’s cellular function. For example, the prognostic relevance of frameshift mutations in the transcription factor CEBPα for patients with acute myeloid leukemia critically depends on which isoform of the protein is truncated [109].

For missense mutations and sequence variants in regulatory regions, genotype/phenotype correlations are even more difficult. One of the genes involved in sensorineural hearing loss is *GJB2,* which encodes a cochlear gap junction ion channel protein called connexin 26 [110]. As the *GJB2* gene is small (one coding exon), detection of sequence variants by Sanger sequencing is relatively straightforward. Since the *GJB2* gene was first implicated in hereditary hearing loss in 1997 [111], over 100 different sequence variants have been described and catalogued in an online database [112]. However, even with *in vitro* assays to elucidate the functional effects of DNA sequence variants on the function of the connexin 26 protein [113], accurately predicting the clinical phenotype remains challenging. If we extend the *GJB2* example to the rest of the genome, comprehensive genotype/phenotype correlation seems difficult if not impossible.

How might clinical molecular diagnostic laboratories tackle the challenge of phenotype prediction in an era of whole-genome analysis? One approach might be to filter whole-genome datasets to enrich for those particular sequence variants that are more likely to be pathogenic. Of course, the first filter is dependent on the comparator, in that a sequence variation present within a particular individual must be defined relative to “reference” individuals who contain their own unique set of sequence variations. As we accumulate sequence data from a large number of individuals across different ethnic backgrounds and health states, our ability to characterize a sequence variation based on population frequency should continue to improve. There are also numerous online databases that collect and annotate SNPs associated with a defined clinical condition. Whole-genome sequence data can also be filtered based on knowledge of protein structure and function. For example, computer algorithms such as PolyPhen [114] that predict the effect of an amino acid substitution on a protein have been used to filter nonsynonymous SNPs discovered through an exome-targeted high-throughput sequencing experiment [32]. Advanced web-based tools such as ProPhylER [115] have also been developed that improve protein structure/function predictions by incorporating additional criteria such as evolutionary constraint. Finally, the most robust filter of SNPs identified through whole-genome sequencing may be through an analysis of the effect of a particular SNP on the dynamic biological networks within the cell. Although annotated online databases of cellular pathways are useful for data mining and gene discovery [116], fully automated approaches to predict the effect of SNPs on biological pathways are still under development [117]. Whereas bioinformatics approaches may someday be the solution to genotype/phenotype correlation, computer algorithms developed to analyze high-throughput sequencing data must be thoroughly validated before they may be applied in clinical diagnostics. 

### 7.3. Clinical utility

For a diagnostic test to impact patient care, the result must directly influence clinical decisions and be communicated to the treating physician in a clear and concise manner. Unfortunately, the complexity of whole-genome datasets does not easily fit within the traditional paradigm of laboratory-based clinical diagnostics. Under the simplest scenario of diagnostic sequence analysis of a gene implicated in a monogenic autosomal recessive disorder, sequence variants can be broadly categorized as a disease-causing mutation, a known polymorphism, or a variant of unknown clinical significance. These simplified descriptors belie a complex synthesis of pathobiology, population genetics, and biochemistry, each modeled with attendant assumptions and bias. If the probabilistic nature of genetic testing results is not appreciated, harm could outweigh benefits because of reactive medicine, resulting in an increased number of screening tests or additional invasive testing. Indeed, the challenge of developing evidence-based scientific standards to evaluate the clinical utility of genomic testing was highlighted in a recent National Institutes of Health multidisciplinary workshop [118]. 

An informative example of the difficulty in applying genomic data to patient care can be drawn from the field of pharmacogenetics. Warfarin is an oral anticoagulant frequently prescribed for patients with thromboembolic disorders. The therapeutic index for warfarin is relatively narrow, and patients must be carefully monitored to prevent bleeding complications. Currently, the optimal dose for a given patient is determined through clinical assessment and repeated laboratory measurement of coagulation status. Genetic polymorphisms in two genes (*CYP2C9* and *VKORC1*) were recently shown to affect patient sensitivity to warfarin [119]. Based on these data, a few small prospective randomized clinical trials [120,121,122] and large retrospective studies [123] have been performed to assess whether pharmacogenetic algorithms could improve warfarin dosing. Despite evidence to suggest that genetic testing may be useful to identify patients who require higher or lower warfarin doses than the mean [123,124], the routine use of pharmacogenetic testing for warfarin dosing remains controversial [125] and is not currently recommended by some professional societies, including the American College of Medical Genetics [126], largely due to the lack of large prospective clinical trials supporting the clinical utility of testing.

Given the apparent difficulty in clinically applying genotype-based risk assessment to a well-defined pharmacogenetic model system, how can we expect to derive accurate and clinically useful risk assessment from the highly complex data sets provided by high-throughput sequencing? Of course, much depends on how the data is gathered. GWA studies using high-throughput sequencing data must be carefully designed and sufficiently powered to detect meaningful gene associations [127] and subsequent meta-analyses of multiple GWA datasets should use uniform inclusion criteria and controls for between-study heterogeneity [128]. Lists of candidate genes identified through these approaches can then be further refined by statistical methods to enrich for functionally related genes within a disease-associated biologic pathway [129]. Despite these efforts, the most accurate calculations of risk will ultimately be derived from randomized controlled prospective clinical trials that evaluate the effect of a particular genotype on clinically relevant outcome measures.

### 7.4. Ethical issues

Genetic testing has always been inexorably intertwined with complex ethical issues. However, the enormity of whole-genome datasets presents new ethical challenges to physicians, patients, and the healthcare system [130]. For clinical laboratory professionals, the key issue involves the analysis and reporting of data. For example, do laboratories have an obligation to report all the sequence variants (including known benign SNPs) that are discovered during whole-genome analysis? Do they have an obligation to re-analyze the data and to provide updated interpretations as new knowledge regarding significance becomes available? How would new information be communicated to patients when risk profiles for disease are changing based on new insights? How could one obtain informed consent when the possible clinical ramifications are not yet fully known or even envisioned? How are the evolving results integrated in the medical record, while protecting data and privacy? These are just a few examples of the plethora of ethical issues that need to be considered and proactively addressed.

## 8. Conclusions

The emergence of next-generation sequencing has opened the door to a new era in diagnostic medicine, bringing the vision of “personalized medicine” closer to reality. As this technology becomes available for health-care applications, physicians and patients will increasingly demand refined diagnosis and treatment strategies tailored to the clinical needs of an individual patient. However, prior to the widespread application of next-generation sequencing for molecular diagnostic testing, several critical processes need to be addressed in a way that results in practical, actionable solutions and effective patient care. This will not only require a multi-disciplinary (inter)national research effort but also a comprehensive translational strategy to apply the data in a clinically meaningful way. Examples of requirements for successful clinical implementation of next-generation sequencing include:

empirical evidence of clinical utility to maximize the benefits and minimize the risk of harm.
profound leap in bio-computational infrastructure and the development of comprehensive programs that aid in the interpretation of massive amounts of genomic data.
standards and laboratory guidelines to help with the clinical interpretation of the results and to facilitate appropriate medical decisions based on this information.
medical students, physicians, laboratory technologists and other health care professionals in these methods.
physicians, patients, and policy makers in the possibilities and limitations of these technologies, as well as the ethical issues surrounding their use.

These are just a few examples of the considerable challenges associated with implementing new sequencing technologies into routine clinical care. However, these barriers can be overcome with concerted effort, prioritization and appropriate resource allocation. To meet the expectations associated with these emerging technologies, diagnostic laboratories may be anticipated to offer more comprehensive sequence analysis than ever before, encompassing the entire genome instead of single genes. However, the most successful initial clinical applications of next-generation sequencing may be through sequencing targeted subsets of the genome, either to identify sequence variants associated with pharmacogenetics, or with inherited and somatic genetic diseases (cancers), by the parallel sequencing of multiple genes or by investigating such changes in candidate regions. Specialized assays to characterize haplotypes, copy number variations, and low numbers of circulating tumor cells or infectious agents will be more widely utilized, and our understanding of infectious diseases should improve through metagenomics approaches. The DNA sequence itself, however, is only one part of an evolving story. More accurate prognostic and diagnostic assays will likely result from our improved understanding of RNA expression (the transcriptome), and epigenetic regulation (DNA binding proteins and chromatin). All these prospects are just emerging, and will require adequate resources and integration of research data before meaningful diagnostic applications will be possible.

Despite current challenges and limitations, reductions in cost and technical advances will undoubtedly enable specialized diagnostic testing laboratories to adopt these technologies in the near future. As genomic information becomes more affordable and readily available, we will witness significant changes in the way medical care is provided and in how patients consider their own life-style choices. The impact of a more comprehensive, proactive, and individualized health care system will be profound, and likely have anticipated as well as unanticipated consequences for patients, physicians, government agencies, insurance providers, and the biotechnology industry. 

In conclusion, the genomic era has begun. However, only when our ability to integrate and responsibly use genomic information parallels our technical capacity to generate it, will we make the long anticipated quantum leap into consequential and widely accessible personalized genomic medicine.
